# Neuromodulation in chronic pain management: addressing persistent doubts in spinal cord stimulation

**DOI:** 10.1186/s44158-024-00219-6

**Published:** 2025-01-06

**Authors:** Giuliano Lo Bianco, Adnan Al-Kaisy, Silvia Natoli, Alaa Abd-Elsayed, Georgios Matis, Alfonso Papa, Leonardo Kapural, Peter Staats

**Affiliations:** 1 Anesthesiology and Pain Department, Foundation G. Giglio Cefalù, Palermo, Italy; 2 Guy’s and St Thomas’ NHS Foundation Trust, The Walton Centre for Neurology and Neurosurgery, Liverpool, UK; 3 Department of Clinical-Surgical, Diagnostic and Pediatric Sciences, University of Pavia, Pavia, 27100 Italy; 4 Pain Unit, Fondazione IRCCS Policlinico San Matteo, Pavia, Italy; 5 Division of Chronic Pain, Department of Anesthesiology, University of Wisconsin School of Medicine and Public Health, Madison, WI USA; 6 Department of Stereotactic and Functional Neurosurgery, Faculty of Medicine and University Hospital, Cologne, Germany; 7 Department of Pain Management, AO “Ospedale Dei Colli”, Monaldi Hospital, Naples, Italy; 8 Carolinas Pain Institute, Winston Salem, NC USA; 9 electroCore, Rockaway, NJ USA; 10 National Spine and Pain Centers, Rockville, MD USA

## Introduction: the evolution of SCS

Over the past 50 years, spinal cord stimulation (SCS) has emerged as one of the most effective treatments for chronic pain. Yet, despite its proven safety and efficacy and lack of adequate alternatives, insurers remain skeptical about the role of SCS. Despite compelling evidence, doubts about this transformative therapy persist, raising questions about the barriers to its broader adoption. Chronic pain is a debilitating condition that affects millions worldwide, diminishing quality of life and productivity. It affects over 30% of individuals globally, and high-impact pain affects 8% of the population. High-impact chronic pain is defined as pain that limits life activities or work on most, if not daily, over the previous 3 months. Despite this, pain is often underdiagnosed and undertreated [1]. For over half a century, SCS has become an effective, non-opioid, chronic pain treatment [2].

**Fig. 1:**
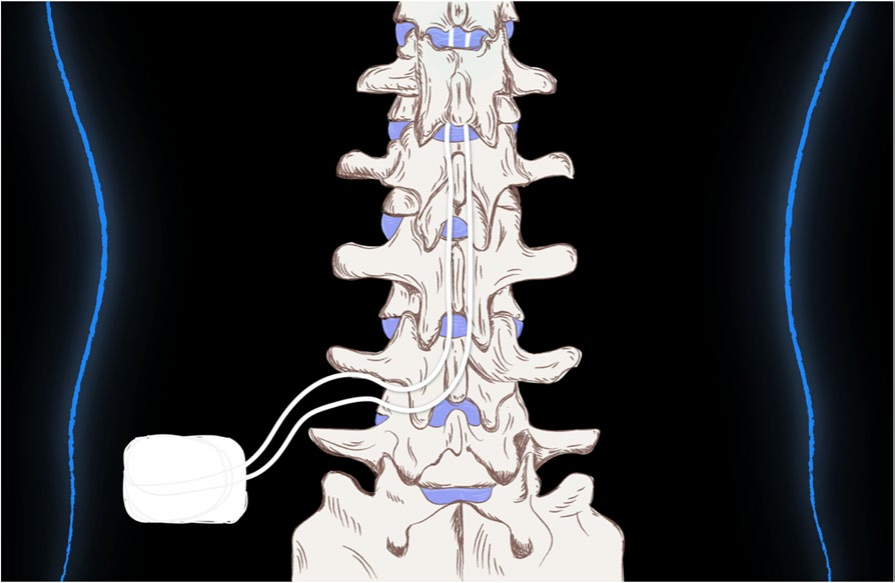
Spinal cord stimulation (SCS) system placement

Traditional treatments, including medications and surgeries, often provide limited relief and come with significant risks and side effects. The number of patients needed to treat (NNT) to achieve more than 50% pain relief success in one patient using conventional pain management is low, from about 4 to 10 [3, 4]! Therefore, the SCS represents a valid alternative, where the NNT ranges from 1.2 to 2 [5] a technique in which a device is implanted near the spinal cord to deliver electrical impulses that modulate neural activity. (Fig. 1).

## Mechanisms of SCS and DRG stimulation

While the precise mechanisms are still debated, SCS modulates pain processing pathways in the spinal cord and brain through several mechanisms. It activates large sensory nerve fibers, such as A-beta fibers, which inhibit pain signals transmitted by smaller, chronic pain-specific fibers like C-fibers and A-delta fibers. Additionally, it influences wide dynamic range neurons (WDR) in the dorsal horn of the spinal cord, reducing hyperexcitability and dampening central sensitization [6]. Beyond this, SCS has been shown to reduce neuroinflammation by inhibiting the activation of glial cells, which play a pivotal role in chronic pain syndromes. These changes can lead to a significant reduction in the perception of pain [6].

Recent advancements have also highlighted the role of dorsal root ganglion (DRG) stimulation in precisely targeting specific pain regions. DRG stimulation directly modulates the primary sensory neurons, allowing for enhanced focal control of pain signals and offering significant relief for conditions such as complex regional pain syndrome (CRPS) and postsurgical groin pain.

## Clinical impact and expanding applications

For many patients, SCS offers a life-changing reduction in pain, sometimes succeeding where other treatments have failed [7]. Utilization of SCS as a treatment option, however, varies significantly among nations and even within them. Despite supportive organizational guidelines, position statements, and a high level of clinical evidence for SCS in treating multiple conditions [7–12], patient referral and reimbursement for advanced treatment are infrequent and often delayed for years after symptom onset and disability development. The effectiveness of SCS is supported by extensive research [2, 7]. The efficacy of SCS is well-established in the management of refractory mixed neuropathic/nociceptive and neuropathic/radicular pain conditions, including persistent spinal pain syndrome (PSPS), complex regional pain syndrome (CRPS), diabetic neuropathy, failed neck surgery syndrome, and low back pain in patients without indication for surgical treatment [2, 3]. Numerous studies have shown significant pain relief in these conditions (Table 1) [2, 7–10]. Clinical trials have demonstrated that SCS not only reduces pain but also decreases the need for opioid medications [13], which is a crucial benefit, especially during the ongoing opioid crisis.

**Table 1:** Indications, common complications, and limitations of spinal cord stimulation (SCS)

***Indications***	• Persistent spinal pain syndrome (PSPS)	Kumar et al., 2008 [14] (RCT), North et al., 2005 [15] (RCT)
	• Complex regional pain syndrome (CRPS)	Kemler et al., 2000 [16] (RCT), de Vos et al., 2014 [17] (RCT)
	• Diabetic neuropathy	Sayed D. et al., 2024 [13]
	• Low back pain (without surgical indication)	Kapural et al., 2023 [18] (RCT), Deer et al., 2024 [12] (RCT), Kallewaard, 2024 [19] (RCT)
	• Refractory mixed neuropathic/nociceptive and neuropathic/radicular pain conditions	Mekhail et al., 2011 [20] (guidelines), Deer et al., 2014 [7, 11] (case series)
***Common complications***	• Lead migration (approximately 5% of cases)	Rauck R. L. et al., 2023 [21]
	• Postdural puncture headache	Kallewaard et al., 2024 [19]
	• Pocket pain (varies based on body habitus, BMI, and type of IPG)	Deer et al., 2024 [12] (guidelines)
	• Tolerance to SCS over time	Kumar et al., 2006 [22] (retrospective study), North et al., 1991 [23] (long-term follow-up)
	• Device malfunction	Mekhail et al., 2011 [20] (guidelines)
***Limitations***	• Lack of blinded, parallel randomized controlled trials (RCTs)	North et al., 2005 [15] (RCT), Kumar et al., 2008 [14] (RCT)
	• Limited evaluation of long-term effects in RCTs	North et al., 1991 [23] (long-term follow-up)
	• Potential bias in current evidence due to reliance on open-label or non-randomized trials	Mekhail et al., 2011 [20] (guidelines)
	• Concerns about the durability of SCS benefits over time	Kumar et al., 2006 [22] (retrospective study), North et al., 1991 [23] (long-term follow-up)
	• Small sample sizes and short follow-up periods in some studies	de Vos et al., 2014 [17] (RCT), Kemler et al., 2000 [16] (RCT)
	• Limited knowledge and familiarity among healthcare providers	Mekhail et al., 2011 [20] (guidelines)

In addition, there is a growing body of evidence indicating that SCS can effectively manage pain in conditions beyond traditional neuropathic pain syndromes. Specifically, studies have shown promising results in using SCS to alleviate chronic abdominal pain, where conventional therapies often fall short [24]. Similarly, SCS has been explored as a treatment option for chronic pelvic pain, providing relief for patients who have not responded to other interventions [24]. Moreover, research is increasingly supporting the use of SCS for refractory headache disorders, such as chronic migraines, where other treatments have failed [25]. These emerging applications of SCS highlight its potential as a versatile tool in managing complex and otherwise intractable pain conditions.

## Limitations and future directions

However, the evidence for spinal cord stimulation has been criticized for the absence of blinded, placebo-controlled randomized controlled trials (RCTs) and limited evaluations of the long-term effects of SCS in RCTs [10]. Much of the research over the past 20 years has been comparative effectiveness trials concentrating on comparing standard of care or comparing multiple frequencies that have previously established efficacy. Critics argue that much of the current evidence relies on open-label studies or non-randomized trials, which are not placebo controlled, which may introduce biases and affect the reliability of the results.

That being said, there have been significant strides in the field, with multiple RCTs conducted across various neuromodulation therapies. Specifically, there are now 3 RCTs for burst stimulation, 1 RCT for closed-loop stimulation, 3 RCTs for 10-kHz high-frequency stimulation, 2 RCTs for dorsal root ganglion (DRG) stimulation, 2 RCTs associated with sub-threshold ≤ 1.2-kHz Boston Scientific devices, and 2 RCTs for differential target multiplexed (DTM) stimulation with Medtronic. These trials represent essential contributions to the growing body of evidence, although the need for more robust, blinded, and long-term studies remains [19, 26–28].

In addition, real-world, long-term data have suggested a possible loss of efficacy over time, raising concerns about the durability of SCS benefits with traditional systems [2].

More recently, advancements in paresthesia-free, novel waveforms and closed-loop stimulation have shown promise in improving long-term patient outcomes over traditional tonic stimulation. Additional studies could compare the effectiveness of therapeutic options [29]. Addressing these research gaps is crucial for establishing a solid scientific foundation for SCS and enhancing its clinical application.

While the benefits of SCS are significant, it is essential to acknowledge and understand the potential complications and limitations associated with this kind of therapy. Lead migration is one of the more common complications, which can occur in up to 5% of cases [2, 21, 30]. Electrode migration can lead to low efficacy as the electrical pulses are no longer directed at the intended area. With early devices, electrode fracture has had a reported prevalence as high as 5.9%, which has significantly improved over time [4, 22, 30]. Pocket pain, another frequent complication, can vary based on body habitus, surgical technique, body mass index (BMI), and implantable pulse generator (IPG) type [5, 31] (Table 1, Fig. 1). Rechargeable IPGs tend to be smaller and may cause less discomfort than non-rechargeable ones, but they require regular maintenance and recharging, which can be inconvenient for some patients [21, 30–32].

Tolerance to spinal cord stimulation (SCS), where the therapy’s effectiveness diminishes over time, is another concern. This phenomenon may occur, from lead fibrosis, changes in pain perception, and neuroplasticity. The most common reason for “tolerance” with traditional stimulation is probably due to repeated episodes of overstimulation that causes patients to turn down the stimulation to a subtherapeutic pattern. Overstimulation can occur with daily activities, from coughing or deep breathing to ambulation, where the activation of the spinal cord changes dramatically [22].

A noteworthy option in neuromodulation is dorsal root ganglion (DRG) stimulation [5, 33]. This technique targets the dorsal root ganglion, a cluster of nerve cell bodies within the spinal cord, which plays a crucial role in transmitting pain signals. DRG stimulation has shown efficacy in treating focal, neuropathic pain conditions that are otherwise difficult to manage with traditional SCS [5, 22, 34]. For instance, it has successfully treated CRPS and groin pain following hernia repair [35]. DRG stimulation offers several advantages over conventional SCS. It allows for more precise targeting of pain areas, leading to potentially greater pain relief, fewer side effects, and lower variability in stimulation intensity [34, 36]. Moreover, DRG stimulation has shown promising results in patients who have not responded well to traditional SCS, making it a valuable option for those with refractory pain conditionsm [37].

However, despite these benefits, DRG stimulation is not without its challenges. There have been reports of complications such as lead fractures and nerve damage, which can complicate treatment and lead to additional interventions. These risks underscore the importance of careful patient selection and meticulous surgical technique to minimize potential adverse outcomes [4–13, 19, 24–30, 38].

## Advancements and accessibility

Advancements in technology have made SCS more accessible and customizable. Modern devices offer adjustable settings tailored to individual patient’s needs, improving outcomes and patient satisfaction [31]. One of the latest advancements includes cutting-edge stimulation techniques such as closed-loop technology, which addresses maintaining optimal stimulation levels during movements. This technology accounts for the dynamic nature of the epidural space, reducing the risk of under or overstimulation and mitigating tolerance issues [26].

Additionally, the industry is increasingly embracing remote monitoring, which allows for continuous, real-time adjustments and follow-up care, further enhancing the effectiveness and convenience of SCS therapy [6, 39].

Some physicians are concerned with the invasiveness of the procedure [40–42]. While SCS involves surgery, it is minimally invasive and much safer compared to many other interventions. The implantation process is typically straightforward, with low risks and quick recovery times. Additionally, trial stimulation periods allow patients to experience the benefits before committing to permanent implantation, reducing the risk of unsuccessful outcomes [43].

Financial concerns also contribute to skepticism [44]. The initial cost of SCS can be high, but it must be weighed against the long-term benefits. Reduced medication dependence, fewer doctor visits, and improved quality of life can save overall costs [45]. Farber et al. [46] also validated the cost-effectiveness of SCS over 10-year post-implantation, despite the relatively high initial cost of implantation. Insurance coverage for SCS has also improved, making it more accessible to a broader patient population [47].

## Challenges and opportunities

Despite the robust clinical evidence, safety, and technological advances, it is estimated that only 5% of patients who are candidates for treatment with neuromodulation ever get access to SCS [48]. Meta-analyses and systematic reviews provide robust evidence supporting the functional benefits of spinal cord stimulation (SCS) compared to conventional medical management or additional spinal surgeries. For instance, a comprehensive meta-analysis reported significant improvements in pain relief and quality of life for SCS patients, with a marked reduction in disability scores such as the Oswestry Disability Index (ODI) and enhancements in physical functioning measured by tools like the SF-36 survey. These studies highlight the efficacy of SCS in reducing chronic pain and improving patient mobility and daily functioning [22, 32, 33, 49].

SCS has emerged as a vital tool in addressing the opioid epidemic by providing a non-pharmacological alternative for pain management with both efficacy and safety, including the ability to reduce opioid use in patients with chronic pain with an incidence of major complications of less than 1% [34, 35, 49, 50].

However, SCS is not suitable for all types of pain and should be considered as part of a broader strategy to combat the opioid crisis. This strategy must include other measures to effectively address this complex issue.

Another hurdle is the conservative nature of medical practice [51]. Physicians may be hesitant to recommend SCS due to a lack of familiarity, biasis, or experience with the technology. Increasing awareness and education about the procedure among healthcare providers is essential. As more doctors witness the transformative effects of SCS in their patients, acceptance will likely grow.

Furthermore, there is a shortage of healthcare professionals who can provide appropriate indications for spinal cord stimulation, likely due to insufficient awareness [52]. Patients with chronic and/or neuropathic pain often do not initially seek help from pain medicine specialists but instead consult other specialists (such as orthopedic surgeons, neurologists, physiatrists, or neurosurgeons). Unfortunately, these specialists may lack the knowledge to recommend spinal cord stimulation correctly. As a result, patients may never be offered appropriate therapy, and their treatment may involve fewer practical approaches, potentially compromising long-term outcomes [53].

Patients’ fear of the unknown and reluctance to undergo surgical procedures can also delay the adoption of SCS [54, 55]. Comprehensive patient education and support are vital in addressing these concerns. Success stories and testimonials from those who have regained their lives through SCS can be powerful motivators [56].

The procedure is often reserved to carefully selected patients, with patient selection being crucial, excluding those with substance abuse issues, widespread pain, or psychological problems. For conditions like persistent spinal pain syndrome type 2 (PSPS2), SCS remains a primary long-term solution for pain relief. Clinical studies indicate that SCS can provide significant pain relief, with > 80% of patients experiencing at least a 50% reduction in pain levels with current technologies [24, 25]. The pain relief achieved with SCS typically lasts for several years, with ongoing effectiveness often contingent upon regular follow-up and management [25].

The efficacy of SCS in treating PSPS2 can vary depending on the specific pain phenotype. For instance, patients with PSPS2 characterized by predominant leg pain often report better outcomes compared to those with persistent low back nociceptive pain. Studies suggest that patients with radicular pain or leg pain may experience more substantial relief, while those with more diffuse or nociceptive low back pain may see less pronounced benefits. As such, patient selection and a tailored approach to treatment are crucial for optimizing outcomes with SCS [7, 57]. Additionally, a stimulation trial helps minimize errors [58–60].

Over the past decade, the indications for SCS have expanded to include PSPS type 1 [58–60]. This type of PSPS refers to chronic lower back pain that persists despite patients undergoing conservative treatments and pain relief procedures without having had prior spine surgery. This condition poses a significant challenge for clinicians, as patients continue to experience disabling pain despite receiving nonsurgical interventions. Moreover, there is limited evidence supporting the effectiveness of spinal fusion or disc replacement surgeries in managing chronic lower back pain in patients without prior surgery. Given the limited effectiveness of treatments such as physical therapy, medications, injections, and nonsurgical interventions in these cases, there is a growing need for alternative therapies [58–60].

Supported by numerous randomized controlled trials (Table 2), SCS offers substantial pain relief, enhances functionality, and prevents unnecessary surgeries, positioning it as an essential tool for the future of chronic pain management [19, 27, 61].

**Table 2:** RCT studies in the management of persistent spinal pain syndrome type 1

Company	Waveform	RCT	No.of patients	Duration	Responder rate
**Medtronic**	DTM	DTM vs SCS^17^	128	12 months	80.1% vs 51.2%
		DTM vs CMM^15^	115	24 months	> 80%
**Nevro**	10 kHz	10 kHz vs CMM^51^	159	18 months	83.1%
**Abbott**	Burst DR	DTM vs CMM^52^	269	6 months	85.1%
**Boston Scientific**	MM therapy	MM vs CMM^5^	128	12 months	88%

In conclusion, SCS offers significant benefits for many patients with chronic pain, including substantial pain relief and a reduction in opioid use. Despite these advantages, SCS has challenges and limitations, such as the need for careful patient selection and variability in treatment outcomes based on pain phenotype. The doubts surrounding SCS stem from legitimate concerns about its long-term efficacy and the need for more comprehensive research to understand its role in pain management better. As the medical community continues to explore and address these issues, it is essential to maintain a balanced perspective. While SCS remains a promising and valuable option in the fight against chronic pain, ongoing scrutiny and refinement of the treatment approach are crucial to maximizing its benefits and minimizing potential drawbacks.

This table outlines the primary indications for spinal cord stimulation (SCS) in the management of chronic pain, including key studies supporting each indication. Additionally, it highlights common complications and known limitations of the therapy. For each category, relevant references are provided to indicate the source of clinical evidence. The table serves to summarize the benefits of SCS while addressing the potential complications and limitations associated with its use, supporting a balanced perspective on its efficacy in chronic pain management.

This image illustrates the placement of a spinal cord stimulation (SCS) system within the human spine. The SCS device includes an implantable pulse generator (IPG) located subcutaneously near the lower back, connected to leads that are positioned along the spinal cord. The leads deliver electrical impulses to the spinal cord, aiming to modulate pain signals and provide relief for chronic pain conditions.

Table 2 summarizes key randomized controlled trials (RCTs) comparing different spinal cord stimulation (SCS) therapies and conventional medical management (CMM) in patients with persistent spinal pain syndrome type 1. The table highlights the companies involved, the waveform technology used, the number of patients, the duration of follow-up, and the responder rates. Notably, all SCS modalities, including DTM, 10 kHz, burst DR, and MM therapy, demonstrated significant responder rates, with values ranging from 80.1 to 88%, indicating their effectiveness in managing this challenging pain condition.

## Data Availability

No datasets were generated or analysed during the current study.

## Glossary

Spinal cord stimulation (SCS): A therapy that uses electrical impulses delivered via an implanted device to modulate nerve activity and reduce chronic pain
Dorsal root ganglion (DRG): A cluster of sensory nerve cell bodies located near the spinal cord that transmits pain signals to the brain
Paresthesia-free waveforms: A type of stimulation that does not produce the tingling sensation (paresthesia) often associated with traditional SCS, enhancing patient comfort
Closed-loop stimulation: A system that automatically adjusts stimulation levels in real time based on the patient’s movements and physiological responses to maintain effective pain control
Persistent spinal pain syndrome (PSPS): A condition characterized by chronic pain that persists after spinal surgery or conservative treatment
Complex regional pain syndrome (CRPS): A chronic pain condition that often affects a limb, typically after an injury or surgery, and is characterized by severe pain and sensitivity
Number needed to treat (NNT): A statistical measure that indicates the average number of patients who need to be treated to achieve one successful outcome
Electrode migration: A complication where the implanted leads of an SCS device move from their original position, reducing treatment efficacy
Neuroplasticity: The ability of the nervous system to adapt and reorganize itself, which can influence the effectiveness of SCS therapy over time
Opioid crisis: A public health issue arising from widespread misuse of opioid medications, emphasizing the importance of non-opioid pain management options like SCS
